# Development and Validation of an Artificial Intelligence Preoperative Planning and Patient-Specific Instrumentation System for Total Knee Arthroplasty

**DOI:** 10.3390/bioengineering10121417

**Published:** 2023-12-13

**Authors:** Songlin Li, Xingyu Liu, Xi Chen, Hongjun Xu, Yiling Zhang, Wenwei Qian

**Affiliations:** 1 Department of Orthopedic Surgery, Peking Union Medical College Hospital, Chinese Academy of Medical Sciences and Peking Union Medical College, Beijing 100010, China; 2 School of Life Sciences, Tsinghua University, Beijing 100084, China; 3 Institute of Biomedical and Health Engineering (iBHE), Tsinghua Shenzhen International Graduate School, Shenzhen 518000, China; 4 Department of Biomedical Engineering, School of Medicine, Tsinghua University, Beijing 100084, China; 5 Departments of Orthopedics, West China Hospital, West China School of Medicine, Sichuan University, Chengdu 610041, China

**Keywords:** knee arthroplasty, artificial intelligence, machine learning, patient-specific instrumentation

## Abstract

Background: Accurate preoperative planning for total knee arthroplasty (TKA) is crucial. Computed tomography (CT)-based preoperative planning offers more comprehensive information and can also be used to design patient-specific instrumentation (PSI), but it requires well-reconstructed and segmented images, and the process is complex and time-consuming. This study aimed to develop an artificial intelligence (AI) preoperative planning and PSI system for TKA and to validate its time savings and accuracy in clinical applications. Methods: The 3D-UNet and modified HRNet neural network structures were used to develop the AI preoperative planning and PSI system (AIJOINT). Forty-two patients who were scheduled for TKA underwent both AI and manual CT processing and planning for component sizing, 20 of whom had their PSIs designed and applied intraoperatively. The time consumed and the size and orientation of the postoperative component were recorded. Results: The Dice similarity coefficient (DSC) and loss function indicated excellent performance of the neural network structure in CT image segmentation. AIJOINT was faster than conventional methods for CT segmentation (3.74 ± 0.82 vs. 128.88 ± 17.31 min, *p* < 0.05) and PSI design (35.10 ± 3.98 vs. 159.52 ± 17.14 min, *p* < 0.05) without increasing the time for size planning. The accuracy of AIJOINT in planning the size of both femoral and tibial components was 92.9%, while the accuracy of the conventional method in planning the size of the femoral and tibial components was 42.9% and 47.6%, respectively (*p* < 0.05). In addition, AI-based PSI improved the accuracy of the hip–knee–ankle angle and reduced postoperative blood loss (*p* < 0.05). Conclusion: AIJOINT significantly reduces the time needed for CT processing and PSI design without increasing the time for size planning, accurately predicts the component size, and improves the accuracy of lower limb alignment in TKA patients, providing a meaningful supplement to the application of AI in orthopaedics.

## 1. Introduction

Total knee arthroplasty (TKA) is an effective treatment for end-stage knee osteoarthritis, rheumatoid arthritis, and osteonecrosis, and its demand is increasing every year [1,2]. Despite the good results achieved with TKA, approximately 20% of patients still report dissatisfaction postoperatively [3,4]. The size and orientation of the component have been shown to be related to postoperative clinical outcomes and longevity; therefore, precise preoperative planning to determine the size and position of the component is essential [5,6]. Precise preoperative planning avoids unpredictable intraoperative challenges and reduces component and instrumentation stock and sterilization costs [7].

Traditional two-dimensional (2D) templating is less accurate in assessing the component size and does not consider factors such as the thickness of the resected bone [8,9]. This is due to inconsistencies in magnification, difficulty in obtaining standard radiographs, complex anatomies in patients, and the two-dimensionality of the X-ray image [10,11]. Computed tomography (CT)-based three-dimensional (3D) preoperative planning can better take these factors into account and has been shown to provide more accurate component size prediction for TKA patients, in addition to providing information on the thickness of resected bone, component orientation, etc. [11,12]. However, it requires good image reconstruction and segmentation, and the whole process is complex and time-consuming, which limits its application [13].

With a basis in CT-based preoperative planning, digital technologies, such as navigation, robot-assisted techniques, and patient-specific instrumentation (PSI), are being used in arthroplasty [14,15,16]. All these techniques have been reported to improve surgical accuracy, but navigation and robotic technologies require costly purchases and maintenance, increase the operating time, and require additional, sufficiently large spaces for application and storage. The PSI is less expensive, requires less space for storage, and has become an attractive tool among surgeons because it does not increase the operating time [7]. However, the traditional PSI design process is complex and requires checking the fit of the PSI to the bone surface at multiple levels, thereby increasing its preparation time, resulting in the entire production process taking 4–8 weeks [17,18].

Artificial intelligence (AI) and machine learning (ML) aim to achieve some form of intelligence, enabling computer systems to perform tasks that typically require human intelligence. They both rely on learning and analysis of data to improve performance. Both are being increasingly used in various fields of clinical medicine [19,20,21]. Because of its effectiveness in image segmentation and handling massive amounts of data, it has been applied in the identification and prediction processes of a wide range of imaging modalities, thus saving time and costs and increasing procedural accuracy [22,23]. In addition, machine learning has been widely applied in 3D printing, encompassing areas such as process optimization and material design [24]. AI and machine learning are well suited for lower extremity arthroplasty due to their elective nature, capacity to meet the needs of centres with high patient volumes, and suitability for shifting payment models. Most applications are currently focused on disease diagnosis [25,26]. There has been previous application of AI preoperative planning combined with PSI in the hip arthroplasty [19], but there has been limited application in TKA to date.

It is necessary to develop a rapid and accurate TKA planning system with the assistance of artificial intelligence that allows image segmentation and reconstruction, identification of feature anatomic landmarks, and planning of component size and orientation as well as PSI design. The objectives of this study were to develop and construct an artificial intelligence-based TKA planning system; compare the time needed for CT processing, component size planning, and PSI design between artificial intelligence and manual methods; and clinically validate its accuracy in planning component size and designing the PSI.

## 2. Materials and Methods

This study was approved by the institutional review board (IRB) of Peking Union Medical College Hospital (I-23PJ842), and an informed consent waiver was approved by the IRB. All procedures performed in this study involving human participants were in accordance with the ethical standards of the institutional and/or national research committee and with the 1964 Helsinki Declaration and its later amendments or comparable ethical standards.

### 2.1. Development and Construction of the Artificial Intelligence Preoperative Planning and Patient-Specific Instrumentation System for Total Knee Arthroplasty (AIJOINT)

AIJOINT (Version 2.0, Longwood Valley Technology, Beijing, China) consists of three modules: CT image processing, component planning, and PSI designing. The CT images are first segmented to identify the femur and tibia, and feature anatomical landmark points are automatically identified to determine the anatomical and mechanical axes of the femur and tibia. The component planning module can be used to plan the size of the component and the thickness of the resected bone, adjust the position of the component in a three-dimensional view, determine the post-osteotomy status, and visualize the coverage after placement of the component. The position and fitting surface of the PSI can also be designed automatically according to the component planning in the PSI design module.

#### 2.1.1. CT Data Acquisition

Over 600 thousand eligible CT images of the lower limbs from 300 anonymous patients were included in this study. All patients were scheduled to undergo TKA. The primary diagnoses included osteoarthritis, rheumatoid arthritis, posttraumatic osteoarthritis, and osteonecrosis. A preoperative standardized CT examination of both lower limbs was performed, and the patient was placed in the supine position with their lower limbs extended as far as possible and their patella directed upwards. The range of each CT scan began from the highest point of the pelvis to the ankle joint at 1 mm intervals (512 × 512 matrix; 120–140 kV; 200–250 mA). All data were stored in DICOM format in a cloud-based database.

#### 2.1.2. Image Segmentation

**Figure 1:**
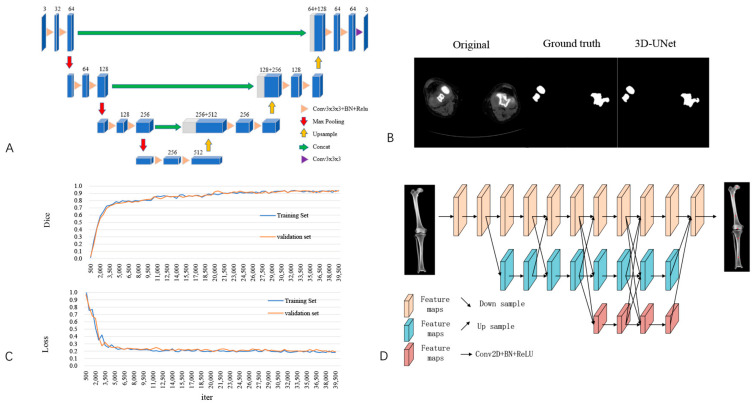
Development of artificial intelligence preoperative planning and patient-specific instrumentation system for total knee arthroplasty (AIJOINT). (**A**) Network structure for image segmentation. (**B**) Segmentation of the femur and tibia. From left to right: images of original computed tomography (CT), manual segmentation, and automatic segmentation with AIJOINT in osteoarthritis. (**C**) Performance of AIJOINT in automatic segmentation. Dice similarity coefficient (DSC) of the training set and validation set. Loss of the training set and validation set. (**D**) Modified HRNet neural network structure used to identify featured anatomic landmarks. The red points represent the following automatic identification: the centres of the femoral head and intercondylar fossa and the medullary midpoints of the femur and tibia.

The complete dataset was randomly assigned to a training set, validation set, and testing set at a ratio of 7:2:1. All images were resized to 512 × 512 pixels. The neural network structure was developed based on the 3D-UNet. The 3D-UNet includes an encoding section and a decoding section. The CT images of the entire sequence are input, and the encoding part contains two 3 × 3 × 3 convolutions per layer, followed by a BN + ReLU, and then 2 × 2 × 2 max pooling with a stride of two. For the decoding part, each layer has a 2 × 2 × 2 up-convolution operation with a stride of two, followed by two 3 × 3 × 3 convolutions and a BN + ReLU. A shortcut connection, similar to a two-dimensional UNet, provides high-resolution features for the decoding layer. In the final layer, a 1 × 1 × 1 convolution reduces the number of output channels to the number of labels and uses softmax as the loss function (Figure 1).

A comparison of the time taken to segment CT images by 3D-UNet versus manual segmentation was performed. CT data from 42 patients who underwent TKA at one hospital between September 2020 and March 2023 were used for both automatic and manual segmentation. Manual segmentation was performed by one orthopaedic attending physician and two trained engineers. The results were reviewed by a chief physician. Cases that did not pass the review were sent back for resegmentation. The time taken for resegmentation was recorded.

#### 2.1.3. Identification of Feature Anatomic Landmarks

The modified HRNet neural network structure was used to identify anatomical feature points, including the centres of the femoral head and intercondylar fossa and the medullary midpoint of the femur and tibia (Figure 1D). These anatomical feature points allow the anatomical and mechanical axes of the femur and tibia, as well as their angulation, to be identified. The modified HRNet began with high-resolution feature images as the first stage, progressively adding high- to low-resolution feature images and transferring the multiresolution feature images in parallel connections. The parallel feature image of the latter stage consisted of the feature image of the previous stage and an additional, lower-resolution feature image, thus enabling the fusion of three different resolution feature layers for output. Compared to the original HRNet, this modification minimizes the loss of features and improves recognition accuracy.

#### 2.1.4. Preoperative Planning Module

**Figure 2:**
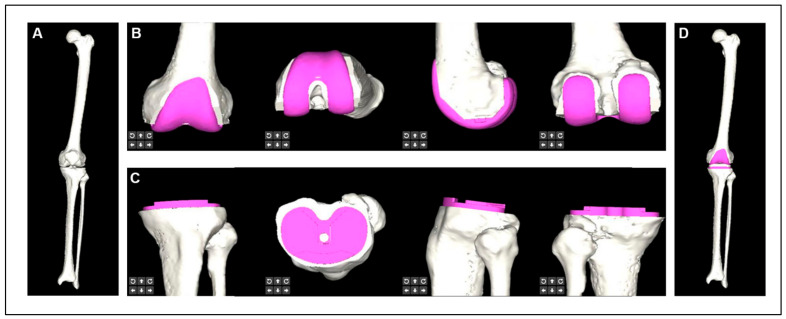
Prosthesis planning module for artificial intelligence preoperative planning and patient-specific instrumentation system for total knee arthroplasty (AIJOINT). (**A**) 3D reconstructed femur, tibia, and fibula. (**B**) Preoperative planning of the femoral component. (**C**) Preoperative planning of the tibial component. (**D**) 3D reconstruction for postoperative implantation.

After CT segmentation and identification, a 3D model of the patient’s bone was generated. The rotation (minimum 0.5°) and translation (minimum 0.5 mm) of the component could be adjusted in the coronal, sagittal, and transverse planes to select the largest component without anteroposterior or mediolateral overhang and to maximize bone coverage while avoiding the notch, posterior cruciate ligament attachment, and osteophyte (Figure 2). Each adjustment allowed real-time observation of the change in thickness of the resected bone and alignment, as well as a three-dimensional simulation of the postoperative effect to select the optimum component size and determine the position of the component depending on the surgeon’s preference for alignment philosophy.

#### 2.1.5. PSI Design Module

**Figure 3:**
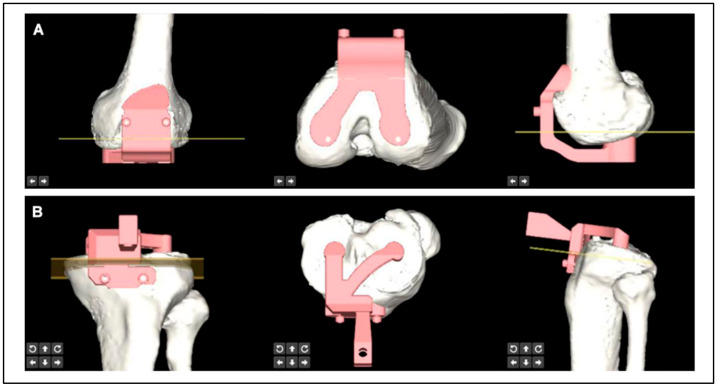
Patient-specific instrumentation design module for artificial intelligence preoperative planning and patient-specific instrumentation system for total knee arthroplasty (AIJOINT). (**A**) Design of the femoral patient-specific instrumentation. The patient-specific instrumentation-guided groove is automatically parallel to the planned osteotomy plane (yellow planes and lines), and the unique fit and shape can be automatically determined. (**B**) Design of the tibial patient-specific instrumentation. The patient-specific instrumentation-guided groove is automatically parallel to the planned osteotomy plane (yellow planes and lines), and the unique fit and shape can be automatically determined.

After the preoperative plan has been determined, the PSI module can be automatically matched to the osteotomy plane, and the unique fit and shape can be determined (Figure 3). The PSI enables osteotomy of both the distal femur and proximal tibia and determines the rotation of the femoral component. The position of the PSI can also be adjusted (medial and lateral adjustment of the femur and medial and lateral, anterior and posterior, and rotational adjustment of the tibia in the horizontal plane) according to the estimated intraoperative exposure without changing the thickness and rotation of the resected bone. The shape of the PSI fitting area can be determined automatically after each adjustment. Thereafter, the engineer could directly post-process the PSI to meet the requirements of printing.

### 2.2. Clinical Validation of the AIJOINT

From September 2020 to March 2023, a total of 42 patients undergoing TKA for osteoarthritis underwent AI preoperative component size planning, and 20 of these patients underwent PSI-guided surgery using AIJOINT. Twenty patients who underwent manual TKA were matched according to age, sex, date of surgery, and surgeon; these matched patients were the conventional group, and there were no significant differences between the two groups in terms of demographic characteristics. See Table 1 for details.

#### 2.2.1. Component Size Planning

Three-dimensional planning was performed preoperatively by an engineer and the surgeon together in accordance with the method mentioned above. The sizes of the AI-planned femoral component and tibial component for 42 patients were recorded. Two attending physicians, who were blinded to the final TKA components retrospectively, templated preoperative anteroposterior and lateral radiographs using acetate templating. The radiographs were first calibrated for magnification using the ball bearings that were visible on the radiographs before templating. All the results were reviewed by a chief physician. The time taken for both methods was recorded.

#### 2.2.2. PSI Design

For the patients in the AIJOINT group, the position of the component and the thickness of the resected bone were planned using a restricted kinematic alignment (rKA) philosophy, which was performed by an engineer and the surgeon. Twenty patients had their PSIs printed preoperatively according to the 3D preoperative plan, along with their femoral and tibial models, so that the area to be fitted could be determined for intraoperative reference. All models were made of polymer polyamides and printed in approximately 8 h using rapid prototyping. They were then sent to our hospital to be sterilized and applied during the operation. The design time, including post-processing of the artificial intelligence PSI, and the time from CT processing to completion of PSI printing were recorded prospectively. Mimics-based PSI planning was retrospectively performed for these patients by an experienced engineer who had not been involved in the design of the artificial intelligence-based PSI. The timing of the mimics-based PSI design process was also recorded.

#### 2.2.3. Surgical Technique

**Figure 4:**
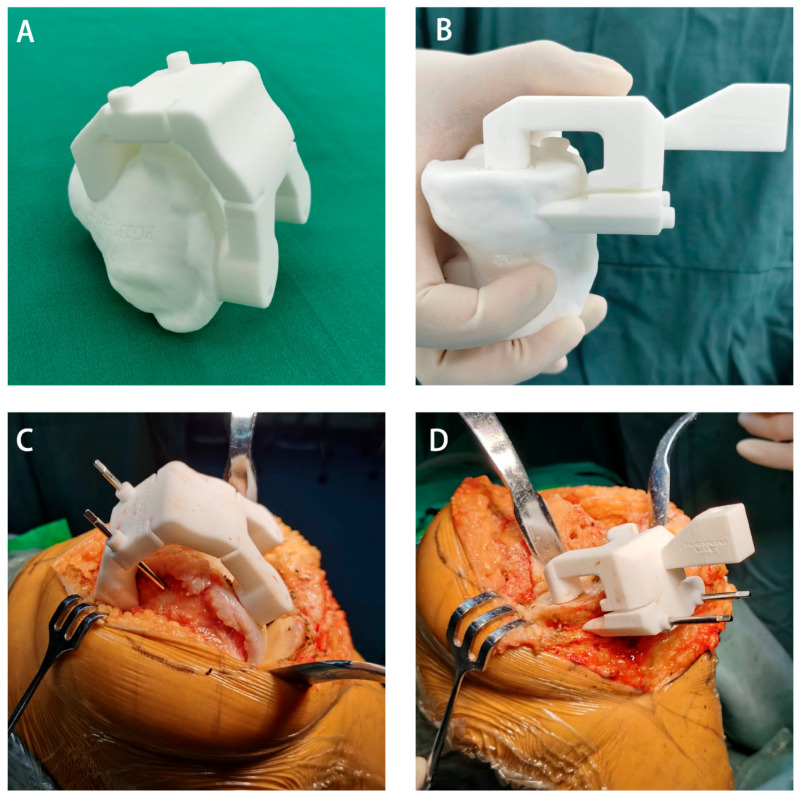
Surgical procedure of artificial intelligence preoperative planning and patient-specific instrumentation system for total knee arthroplasty (AIJOINT). (**A**) Identification of the fitting zones of the custom cutting guides on the femoral bone model. (**B**) Identification of fitting zones of the custom cutting guides on the tibial bone model. (**C**) Intraoperative view of the application of the patient-specific instrumentation on the femoral side. (**D**) Intraoperative view of the application of the patient-specific instrumentation on the tibial side.

All operations were performed by a surgeon with many years of experience in performing knee arthroplasty. After general anaesthesia, a tourniquet was applied to the affected limb. A medial parapatellar approach was used. The synovial membrane, remaining anterior cruciate ligament, menisci, and part of the fat pad were removed. The CT-based PSI did not account for the thickness of the articular cartilage, and a special curette was used to remove articular cartilage before the PSI was fixed to its unique position (Figure 4). The AIJOINT group underwent osteotomy of the distal femur and proximal tibia under the PSI-guided groove, and the two distal pinholes used to fix the femoral PSI allowed the rotation of the femoral component to be determined. A 4-in-1 cutting block was then inserted into the distal pinholes for resection of the anterior and posterior condyle and chamfer. In the conventional group, the osteotomy was completed using conventional instrumentation in accordance with the principle of mechanical alignment (MA). The appropriate size of the component was selected intraoperatively with reference to the coverage, cortical contact, and flexion/extension gap. All the components were cemented cruciate-retaining types. The tourniquet was released after the bone cement had solidified. Standard analgesia, anticoagulant therapy, infection prevention measures, and rehabilitation strategies were applied to all patients postoperatively. Full-length weight-bearing radiographs of both lower limbs were taken once the patient was able to straighten the knee.

#### 2.2.4. Radiographic and Clinical Outcomes

The medial proximal tibial angle (MPTA), lateral distal femoral angle (LDFA), and hip–knee–ankle angle (HKA) were measured postoperatively as previously described and compared to the planned values [12,27]. Measurements were taken and recorded by two observers who were not involved in the surgery, and patient information was anonymized. The mean of the two measurements was used for statistical analysis. The absolute differences between the planned and actual LDFA, MPTA, and HKA were defined as the outline value. The two observers assessed the final TKA component position as described by Peek et al.: tibial components were evaluated to determine the presence of lateral overhang. Femoral components were evaluated according to <50% cortical contact, anterior femoral notching, anterior femoral gap > 2 mm, and posterior condylar contour not restored [28]. The duration of tourniquet use, length of stay, and decrease in haemoglobin values were also recorded. Adverse events included anaemia, deep venous thrombosis (DVT), incision complications, infection, and complications related to pins.

### 2.3. Data Analyses

Statistical analysis was performed with SPSS version 25 (IBM, New York, NY, USA) and GraphPad Prism version 8 (GraphPad Software, San Diego, CA, USA). The Dice similarity coefficient (DSC) and loss function were used to assess the model performance of AIJOINT in the segmentation of CT images. DSC and loss were calculated every 1000 iterations. All data are reported using standard descriptive statistics, including the mean ± standard deviation for continuous variables and count for categorical variables. The outlier of the axis was defined as the difference between the planned component position and the postoperative component position. Continuous variables were compared by the two-sample *t* test or the Wilcoxon rank-sum test. Categorical variables were compared using the chi-squared test or Fisher’s exact test. The interobserver reliability of the measurements by 2 independent investigators was measured using the intraclass correlation coefficient (ICC). A *p* value < 0.05 was considered statistically significant.

## 3. Results

### 3.1. Validation of Artificial Intelligence Algorithms

The DSC and loss curves from the training set and validation set are shown in Figure 1C. The DSC curves for the training and validation reached convergence in 28,500 and 29,500 iterations, respectively. At 28,500 iterations, the DSC of the training set was 0.901, and the DSC of the validation set was 0.913 at 29,500 iterations. Both loss curves reached convergence by 15,800 iterations, and the losses were 0.226 and 0.225 for the training set and validation set, respectively (Figure 1C).

**Figure 5:**
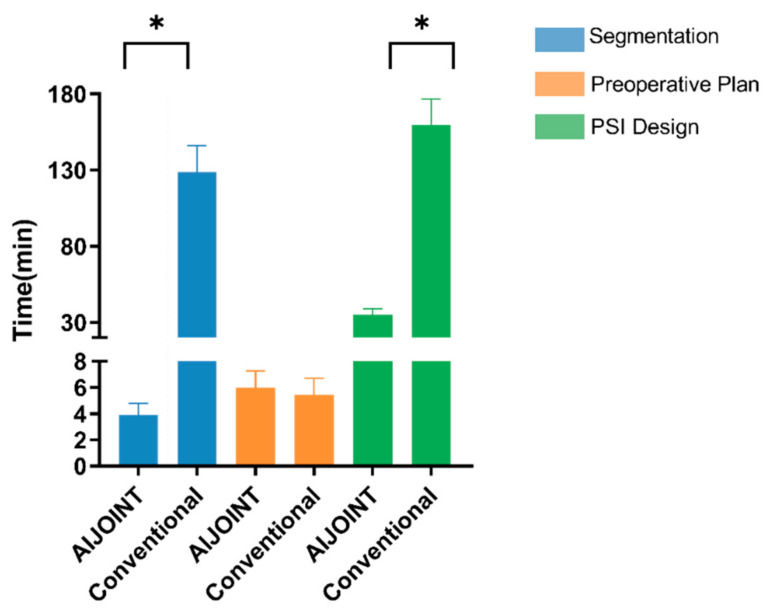
Time comparison between artificial intelligence (AI) processing and manual processing. AI was faster than conventional methods for CT segmentation and PSI design (*p* < 0.05) without increasing the time for size planning. * *p* < 0.001.

The average time to segmentation with artificial intelligence was 3.74 ± 0.82 min and 128.88 ± 17.31 min for manual segmentation (*p* < 0.001). The time needed for AI-based component size planning was 5.98 ± 1.30 min compared to 5.42 ± 1.27 min for acetate templating (*p* > 0.05). In addition, the time needed to design the PSI was 35.10 ± 3.98 min for the AIJOINT and 159.52 ± 17.14 min for the conventional group (*p* < 0.001). The time comparison between AIJOINT processing and conventional processing are shown in Figure 5. The time from CT processing to the completion of PSI printing was 19.86 ± 2.44 h.

### 3.2. Accuracy of 3D and Acetate Templating Compared with the Final Component

No femoral or tibial component met the criteria for poor component positioning. There was no observed lateral overhang of the tibial component or reported femoral components < 50% cortical contact, anterior femoral notching, anterior femoral gap > 2 mm, or posterior condylar contour that was not restored. The size of the actual component can therefore be used as the gold standard. The accuracies of AIJOINT and acetate templating for the femoral component were 92.9% and 42.9%, respectively (*p* < 0.001). The accuracies of AIJOINT and acetate templating for the tibial component were 92.9% and 47.6%, respectively (*p* < 0.001). For the AIJOINT group, in cases where the template did not correctly predict the final component size, the margin of error was never more than one size up or down. See Table 2 for details.

### 3.3. Accuracy of PSI-Assisted Component Positioning

The outlier for the LDFA in the AIJOINT group and conventional group were 1.45 ± 1.70° versus 2.20 ± 1.96°, with 18 and 16 patients, respectively, having outliers within ±3° (*p* > 0.05). The outlier of the MPTA in the AIJOINT group was 1.60 ± 1.82°, with 17 patients having a variance within ±3°. The mean outlier of the MPTA in the conventional group was 2.65 ± 1.84°, with 15 patients having a variance within ±3°. However, the differences were not statistically significant (*p* > 0.05). The mean outlier of the HKA in the AIJOINT group and conventional group was 1.55 ± 1.43° versus 3.35 ± 2.56° (*p* < 0.01), with 18 and 10 patients (90%), respectively, having outliers within ±3°. See Table 3 for details. The ICCs of the LDFA (0.93; 95% confidence interval (CI), 0.86–0.96), MPTA (0.82; 95% CI, 0.69–0.90), and HKA (0.82; 95% CI, 0.67–0.90) were satisfactory (ICC > 0.75).

### 3.4. Perioperative Outcomes

There was no significant difference between the two groups in terms of tourniquet time (65.10 ± 6.77 min vs. 74.55 ± 5.86 min, *p* > 0.05) or length of stay (8.15 ± 1.35 days vs. 7.60 ± 2.06 days, *p* > 0.05). The Hb decrease was 13.50 ± 5.78 g/L in the AIJOINT group and 18.85 ± 10.32 g/l in the manual group (*p* < 0.05). There were no pin-related complications in the AIJOINT group, but delayed incision healing was observed in one patient in the manual group. No thrombosis or infectious complications occurred in either group (*p* > 0.05). See Table 4 for details.

## 4. Discussion

We developed an artificial intelligence preoperative planning and PSI system for TKA (AIJOINT) that can accurately predict component size, and in addition, its designed PSI can improve the accuracy of component orientation and reduce blood loss. AIJOINT was significantly faster than the manual methods in terms of CT processing, component size planning, and PSI design. The overall time from CT processing to PSI printing was less than 24 h.

Accurate segmentation of CT data is essential for the preoperative planning of TKA, which can provide references for the anatomical landmarks and axes of the knee. In addition, manual processing of CT image data is time-consuming and laborious, which affects the efficiency of CT-based preoperative planning. With the development of artificial intelligence and machine learning for the automatic segmentation of medical images, various algorithms have effectively improved segmentation efficiency [22,29], and a convolutional neural network of 3D-UNet was applied in this study. A 3D-UNet performs well for medical image segmentation because it retains better detail and edge information when processing large medical images. The convolutional neural network segmentation model treats each patient’s CT image as an array unit, utilizing GPU acceleration to simultaneously process multiple layers of data. In contrast, manual segmentation requires layer-by-layer segmentation, resulting in lower efficiency [30,31]. Unlike traditional 2D convolutional neural networks, the 3D convolutional neural network employed in this study accounts for spatial and contextual information between CT slices. This enables the model to capture three-dimensional feature information more accurately, leading to more precise segmentation results. In this study, the 3D-UNet-based algorithm significantly improves the speed and accuracy of segmentation. For the identification of feature anatomic landmarks, the modified HRNet enables the output of multiple feature layers with different resolutions to be fused. The loss of features can be reduced to a greater extent, and recognition accuracy can be improved.

The appropriate component size for patients undergoing total knee arthroplasty is critical to postoperative function and long-term survival. An oversized femoral component can cause overstuffing of the patellofemoral joint, affecting knee flexion and patellar tracking and causing anterior knee pain [32,33]. Undersizing the femur may result in femoral notching, which will increase the risk of periprosthetic fracture [34]. An oversized tibial component could cause irritation of the knee tendons and ligaments, causing postoperative pain, while an undersized tibial component could cause subsidence of the tibial tray [35]. Thus, precise preoperative planning is essential for a safe and effective outcome and has been shown to reduce the operative time and sterilization cost [36]. However, preoperative planning can also be challenging. Conventional two-dimensional preoperative planning has accuracy rates as low as 28 to 48% for femoral components and 37 to 55% for tibial components [8,9]. The reason for this may be related to magnification inconsistencies, difficulty in obtaining standard radiographs, complex anatomies in patients, and the two-dimensionality of X-ray images. CT-based 3D planning provides an arbitrary viewing perspective, allowing the position of the component to be viewed dynamically and adjusted in multiple dimensions. Information such as the thickness of the resected bone and the angle of the components can also be provided. Pietrzak et al. found that preoperative three-dimensional templating for robot-assisted TKA was more accurate than two-dimensional digital templating [11]. Preoperative femoral component 3D templating and retrospective blinded two-dimensional templating accuracies were 96.6% and 52.9%, respectively. The tibial component 3D and two-dimensional templating accuracies were 93.1% and 28.7%, respectively. The results of our study were similar to these results. In our study, the accuracy of the AI-based femoral and tibial components was 92.9%, which was significantly better than that of acetate templating. In the AIJOINT software, the simulation of the size and orientation of the component and its relationship to the surrounding bone can be visualized in the coronal, sagittal, and axial planes, allowing the surgeon to adjust the plan in more dimensions.

Accurate lower limb alignment reduces uneven wear of polyethylene bearings and aseptic loosening of the component, prolongs component survival, and positively affects postoperative clinical outcomes [37,38]. The incidence of misalignment of the component after manual TKA has been reported to be as high as 20–30% [39,40]. Various digital technologies for orthopaedic surgeries, such as navigation, robot-assisted surgery, and PSI, have been used to improve the accuracy of component placement. Navigation and robot-assisted surgery can significantly improve surgical accuracy, but they are also disadvantageous in that they are expensive and prolong the operation time, so their generalizability is somewhat limited [14,15]. There is still controversy regarding the use of PSI in improving the accuracy of component alignment. Vide and Huijbregts et al. have shown that a PSI significantly improved osteotomy accuracy when compared to conventional TKA techniques [41,42]. It has also been suggested that PSI-TKA does not differ significantly from conventional TKA in terms of coronal and sagittal alignment, particularly on the tibial side [17,43,44]. We also found that a PSI was only advantageous for the overall lower limb alignment but not on the femoral or tibial side. The reasons for this may be related to the small sample size of the study and that the PSI was not perfectly matched due to the influence of soft tissue and residual cartilage, so further improvement of the design of the PSI is still needed in the future. However, compared to conventional surgery, surgery with a PSI can reduce the operative time and blood loss [45,46]. In addition, complex CT processing and PSI design have long processing times, even requiring 4–8 weeks of preoperative preparation [17,18]. However, in our study, with the help of artificial intelligence and machine learning for CT segmentation and preoperative planning, this process can be reduced to less than 24 h.

The limitations of this study are as follows: 1. First, despite our use of cohort matching, potential bias remains due to the inherent limitations of a retrospective study. 2. The sample size is small, which limits our ability to find discrepancies in some indicators, such as the accuracy of the femoral or tibial component orientation. 3. The alignment philosophy that was used differed between the two groups. However, MA is the gold standard for alignment and is easier to achieve the target alignment manually. 4. We only evaluated the alignment on the coronal plane, and alignment was not evaluated on the sagittal and transverse planes due to the incompleteness of the data. 5. Due to the limitations of the retrospective study, the functional outcomes of the patients were not recorded.

## 5. Conclusions

The artificial intelligence-based AIJOINT addresses the long-standing issue of excessive design time for 3D-printed patient-specific instrumentation in knee arthroplasty, with an overall time from CT processing to 3D-printed patient-specific instrumentation of less than 24 h. AIJOINT accurately predicts the component size and improves the accuracy of lower limb alignment in TKA patients. As artificial intelligence has been increasingly used in the field of medicine, there is an increasing demand for precision, intelligence, and individualization in arthroplasty, and AIJOINT is a significant complement. In addition, 3D-printed metal implants or biodegradable implants might also benefit from this technique in the future.

## Figures and Tables

**Table 1:** Demographic characteristics.

	AIJOINT Group (*n* = 20)	Conventional Group (*n* = 20)	*p* Value
Age (years)	67.95 ± 5.65	69.90 ± 4.71	0.643
Sex (men/women)	6/14	6/14	1.000
BMI (kg/m^2^)	25.11 ± 3.53	25.06 ± 3.09	0.630
Side (left/right)	9/11	9/11	1.000
ASA score	2.10 ± 0.72	2.05 ± 0.61	0.618

BMI: body mass index; ASA: American Society of Anaesthesiologists.

**Table 2:** Comparison of predicted prosthesis size and implanted prosthesis size.

	AIJOINT Group (*n* = 42)	Conventional Group (*n* = 42)	*p* Value
Femoral Component Size Between Preoperative Planning and Postoperative Results (*n*,%)			
Same	39 (92.9%)	18 (42.9%)	0.001
±1 size	42 (100%)	27 (64.3%)	0.001
±2 sizes	42 (100%)	37 (88.1%)	0.055
Tibial Component Size Between Preoperative Planning and Postoperative Results (*n*,%)			
Same	39 (92.9%)	20 (47.6%)	0.001
±1 size	42 (100%)	28 (66.7%)	0.001
±2 sizes	42 (100%)	36 (85.7%)	0.026

**Table 3:** Comparison of planned prosthesis position and implanted prosthesis position.

	AIJOINT Group (*n* = 20)	Conventional Group (*n* = 20)	*p* Value
Outlier of LDFA (°)	1.45 ± 1.70	2.20 ± 1.96	0.204
Outlier of MPTA (°)	1.60 ± 1.82	2.65 ± 1.84	0.078
Outlier of HKA (°)	1.55 ± 1.43	3.35 ± 2.56	0.010
Outlier of LDFA ≤ 3°(*n*, %)	18(90.0%)	16(80.0%)	0.661
Outlier of MPTA ≤ 3°(*n*, %)	17(85.0%)	15(75.0%)	0.695
Outlier of HKA ≤ 3°(*n*, %)	18(90.0%)	10(50.0%)	0.014

LDFA: lateral distal femoral angle; MPTA: medial proximal tibial angle; HKA: hip–knee–ankle angle.

**Table 4:** Surgical-related parameters.

	AIJOINT Group (*n* = 20)	Conventional Group (*n* = 20)	*p* Value
Tourniquet time (min)	65.10 ± 6.77	74.55 ± 5.86	0.719
Length of stay (days)	8.15 ± 1.35	7.60 ± 2.06	0.491
Hb decreased (g/L)	13.50 ± 5.78	18.85 ± 10.32	0.029
DVT (*n*)	0	0	1.000
Incision complications (*n*)	0	1	0.999
Infection (*n*)	0	0	1.000
Pin-related complications(*n*)	0	-	-

Hb: haemoglobin; DVT: deep vein thrombosis.

## Data Availability

The data associated with the paper are not publicly available but are available from the corresponding author upon reasonable request.
